# Dinor-12-oxo-phytodienoic acid conjugation with amino acids inhibits its phytohormone bioactivity in *Marchantia polymorpha*

**DOI:** 10.1093/plphys/kiae610

**Published:** 2024-11-08

**Authors:** Wenting Liang, Ángel M Zamarreño, Salvador Torres-Montilla, Antonio de la Torre, Jean Chrisologue Totozafy, Takuya Kaji, Minoru Ueda, Massimiliano Corso, José M García-Mina, Roberto Solano, Andrea Chini

**Affiliations:** Plant Molecular Genetics Department, National Centre for Biotechnology (CNB), Consejo Superior de Investigaciones Cientificas (CSIC), 28049 Madrid, Spain; Department of Environmental Biology, Bioma Institute, University of Navarra, Navarra 31008, Spain; Plant Molecular Genetics Department, National Centre for Biotechnology (CNB), Consejo Superior de Investigaciones Cientificas (CSIC), 28049 Madrid, Spain; Plant Molecular Genetics Department, National Centre for Biotechnology (CNB), Consejo Superior de Investigaciones Cientificas (CSIC), 28049 Madrid, Spain; Université Paris-Saclay, INRAE, AgroParisTech, Institut Jean-Pierre Bourgin for Plant Sciences (IJPB), 78000 Versailles, France; Graduate School of Science, Tohoku University, Sendai 980-8578, Japan; Graduate School of Science, Tohoku University, Sendai 980-8578, Japan; Université Paris-Saclay, INRAE, AgroParisTech, Institut Jean-Pierre Bourgin for Plant Sciences (IJPB), 78000 Versailles, France; Department of Environmental Biology, Bioma Institute, University of Navarra, Navarra 31008, Spain; Plant Molecular Genetics Department, National Centre for Biotechnology (CNB), Consejo Superior de Investigaciones Cientificas (CSIC), 28049 Madrid, Spain; Plant Molecular Genetics Department, National Centre for Biotechnology (CNB), Consejo Superior de Investigaciones Cientificas (CSIC), 28049 Madrid, Spain

## Abstract

Jasmonates (JAs) are important phytohormones that regulate plant tolerance to biotic and abiotic stresses, and developmental processes. Distinct JAs in different plant lineages activate a conserved signaling pathway that mediates these responses: dinor-12-oxo-phytodienoic acid (dn-OPDA) isomers in bryophytes and lycophytes, and JA-Ile in most vascular plants. In many cases, the final responses triggered by these phytohormones depend on the accumulation of specialized metabolites. To identify compounds regulated by the dn-OPDA pathway in the liverwort *Marchantia polymorpha*, untargeted metabolomic analyses were carried out in response to wounding, a stress that activates the dn-OPDA pathway. A previously unreported group of molecules was identified from these analyses: dn-OPDA-amino acid conjugates (dn-OPDA-aas). Their accumulation after wounding and herbivory was confirmed by targeted metabolic profiling in *Marchantia* and in all species in which we previously detected dn-*iso*-OPDA. Mutants in *GRETCHEN-HAGEN 3A* (Mp*GH3A*) failed to accumulate dn-OPDA-aa conjugates and showed a constitutive activation of the OPDA pathway and increased resistance to herbivory. Our results show that dn-*iso*-OPDA bioactivity is reduced by amino acid conjugation. Therefore, JA conjugation in land plants plays dichotomous roles: jasmonic acid conjugation with isoleucine (Ile) produces the bioactive JA-Ile in tracheophytes, whereas conjugation of dn-*iso*-OPDA with different amino acids deactivates the phytohormone in bryophytes and lycophytes.

## Teaser

Dinor-12-oxo-phytodienoic acid (dn-*iso*-OPDA) conjugates occur naturally, and dn-iso-OPDA conjugation with amino acids reduces its bioactivity in the liverwort *Marchantia polymorpha*.

## Introduction

Jasmonates (JAs) are key regulators of plant development and adaptation to environmental stress, including defense against herbivores and necrotrophic pathogens (Howe et al. 2018; Gasperini and Howe 2024). Studies on *Arabidopsis thaliana*, defined the (+)-7-*iso*-JA-Ile as the ligand of the JA co-receptor complex formed by the F-box CORONATINE INSENSITIVE 1 (COI1) and the jasmonate ZIM domain (JAZ) repressors (Xie et al. 1998; Chini et al. 2007; Thines et al. 2007; Katsir et al. 2008; Fonseca et al. 2009; Sheard et al. 2010). In contrast, studies in *Marchantia polymorpha* identified dn-*iso*-OPDA and Δ^4^-dn-*iso*-OPDA, collectively referred here as dn-OPDAs, as the ligands of a conserved COI1/JAZ co-receptor (Monte et al. 2018; Kneeshaw et al. 2022). Recent studies showed that dn-OPDAs are the bioactive hormones in bryophytes and lycophytes, whereas JA-Ile is the hormone in most vascular plants, except some lycophytes (i.e. lycopodiales) (Monte et al. 2022; Chini et al. 2023). Ligand binding specificity depends on a single amino acid in COI1 that alters the binding pocket size between vascular plants and bryophytes, and on specific residues within the JAZ degron of the JAZ co-receptors (Bowman et al. 2017; Monte et al. 2018, 2022).

The perception of the bioactive JA induces the degradation of the JAZ repressors, which allows the activation of several transcription factors regulating downstream JA-dependent responses (Chini et al. 2007, 2009, 2016; Thines et al. 2007; Sheard et al. 2010; Fernández-Calvo et al. 2011; Monte et al. 2018, 2019; Peñuelas et al. 2019). Recently, an additional signaling role independent on COI1 has been described for the *cis* isomers of dn-OPDA and OPDA. These compounds are reactive electrophilic species that regulate thermotolerance, among other abiotic stresses, in different streptophyte plants, including the charophyte *Klebsormidium nitens*, the liverwort *M. polymorpha* or the angiosperm *A. thaliana* (Farmer and Davoine 2007; Monte et al. 2020).

The JAs biosynthetic pathway is fairly well understood. The octadecanoid and hexadecanoid pathways lead to the formation of OPDA and dn-OPDA, respectively, that are precursors of the bioactive JA-Ile in angiosperms (Chini et al. 2018; Wasternack and Feussner 2018; Yi et al. 2024). In contrast, in *Marchantia* MpFAD5 (FATTY ACID DESATURASE 5) and MpDES6 (Δ^6^-desaturase) are respectively required for the biosynthesis of the hexadecatrienoic acid (16:3) and eicosapentaenoic acid (20:5), major precursors of the bioactive dn-OPDA and Δ^4^-dn-OPDA (Keenshaw et al. 2022; Soriano et al. 2022). In Arabidopsis, the final step in the biosynthesis of the bioactive JA-Ile is mediated by JAR1/GH3.11 (JASMONATE RESISTANT 1) and, to a lesser extent, by GH3.10 that conjugate JA to Ile (Staswick and Tiryaki 2004; Delfin et al. 2022). The JAR1 enzyme and function are well conserved in several vascular plants (Jez 2022; Chini et al. 2023). In addition, the conjugation of JA with amino acids other than Ile has been reported in several plants (Gutierrez et al. 2012). For example, JA-Ala, JA-Val, JA-Leu, and JA-Met are natural molecules whose biosynthesis is induced by wounding in Arabidopsis, tomato, and rice (Suza et al. 2010; Yan et al. 2016). These JA-aa molecules have been described as COI1-JAZ ligands with different affinity to the receptor compared with JA-Ile in angiosperm plants, such as Arabidopsis and tomato (Katsir et al. 2008; Fu et al. 2022).

GH3 enzymes can also catalyze the conjugation of amino acids with other phytohormones such as the auxin indole-3-acetic acid (IAA) and salicylic acid (SA) (Staswick et al. 2005; Westfall et al. 2012; Brunoni et al. 2020, 2023; Jez 2022). IAA-aa conjugates were described as inactive and they were proposed as IAA storage forms (LeClere et al. 2002; Rampey et al. 2004). Plant *GH3* genes usually belong to a large gene family and their extensive functional redundancy hampers to define the precise role of individual enzymes (Gutierrez et al. 2012; Casanova-Sáez et al. 2022).

To identify still unknown compounds regulated by dn-OPDA in *Marchantia*, we carried out untargeted metabolomic analyses. dn-OPDA-aa conjugates were identified as an uncharacterized set of evolutionarily conserved compounds significantly accumulated in response to wounding. To study the biological role of these molecules, we studied the loss-of-function mutants of the Mp*GH3A* gene and found to be required for dn-OPDA conjugation with amino acids. Physiological, transcriptional, and metabolic analyses revealed that Mp*gh3a^ge^* mutants showed a constitutively activated dn-OPDA pathway, suggesting that the activity of dn-*iso*-OPDA is diminished by its conjugation with amino acids.

## Results

### Untargeted metabolomics identify dn-OPDA-amino acid conjugates in *M. polymorpha*

**Figure 1.**
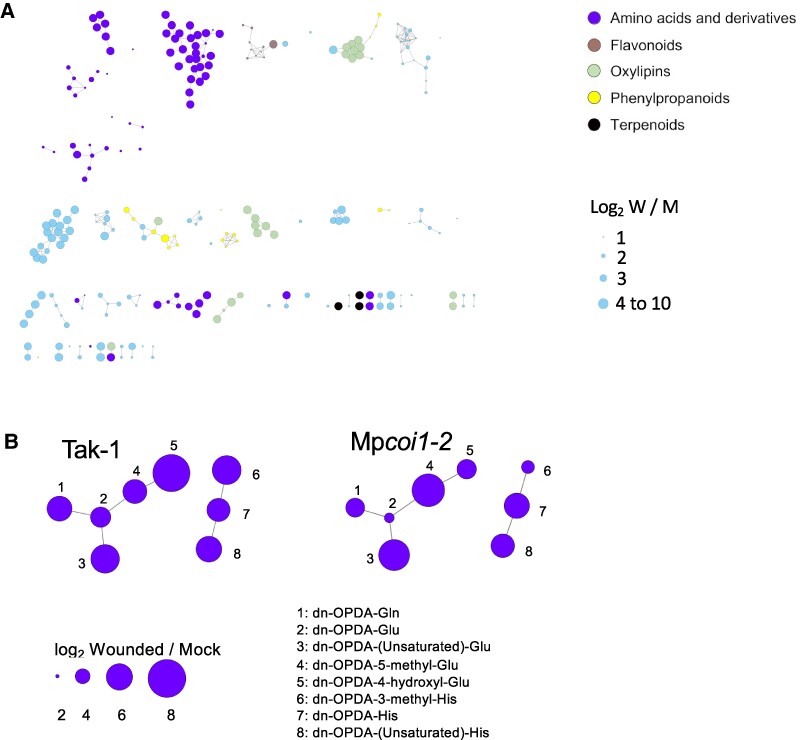
Molecular networks and annotation of *Marchantia* untargeted metabolomic profiles after wounding. **A)** Molecular networks for metabolites differentially accumulated in WT (Tak-1) *Marchantia* after wounding. Analyses were carried out in positive ionization mode (ESI+) ionization modes. Metabolites are grouped based on their chemical structures and different colors correspond to different metabolic classes. The size of the nodes corresponds with the accumulation promoted by wounding (log_2_ wound/mock). Cosine similarity scores of 0.7 were used for ESI+ analysis. **B)** Comparison of molecular network for wound-induced putative dn-OPDA-aa conjugates class in WT (Tak-1) and Mp*coi1-2* mutant plants. The putative identity for each node is shown. The size of the nodes corresponds with the induced accumulation upon wounding (log_2_ wound/mock).

To identify metabolites regulated by dn-OPDA in *M. polymorpha* we carried out untargeted metabolomics analyses of mock and wounded plants using liquid chromatography-mass spectrometry (LC-MS/MS) of crude extracts of WT (Tak-1) and Mp*coi1-2* mutant plants. In addition to the expected classes of JA-regulated molecules previously identified in response to wounding in several plants, such as oxylipins, terpenoids, phenylpropanoids, and flavonoids, we also identified a significant accumulation of a strikingly abundant class of unknown metabolites, putatively annotated as N-acyl amino acids derivatives (Fig. 1A). Further analysis of their putative structure based on MS/MS spectra revealed that some of these compounds could be isomeric forms of dn-OPDA conjugated to glutamic acid, histidine, glutamine, and different methyl- or hydroxyl-derivatives (Supplementary Fig. S1). These naturally occurring molecules had not been described to date. The wound-induced accumulation of putative dn-OPDA amino acid (dn-OPDA-aa) conjugates was slightly diminished in Mp*coi1-2* mutant, suggesting only a minor requirement of the COI1 pathway for their accumulation (Fig. 1B).

**Figure 2.**
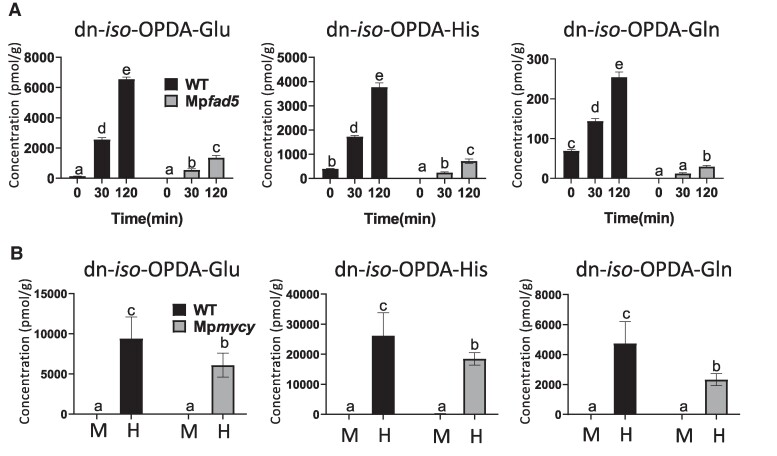
Accumulation of dn-*iso*-OPDA conjugates upon stress in *M. polymorpha*. Time-course accumulation of dn-*iso*-OPDA conjugates [pmol/fresh weight (g)] in WT (Tak-1) *Marchantia* plants, Mp*fad5* or Mp*mycy-1* mutants after wounding **A)** or herbivory challenge **B)**. Plants were wounded and damaged tissues were collected after the indicated times (A). For herbivory assay, plants were challenged with *S. exigua* larvae and damaged tissues (H) were collected 8 days later (B). Unwounded (0) or not-challenged (mock, M) plants were included as control. Data shown as mean ± SD of three biological replicates. Experiments were repeated three times with similar results. Letters indicate significant different samples according to the one-way ANOVA/Tukey HSD post hoc test (*P* < 0.05).

To validate the ability of *Marchantia* plants to synthetize dn-OPDA-aa compounds after wounding, we analyzed the accumulation of dn-OPDA conjugates with all natural amino acids in targeted LC-MS analyses. Nine dn-OPDA-aa conjugates were detected and most of them significantly accumulated in response to wounding (Supplementary Figs. S1 and S2). To confirm the identity of these dn-OPDA-aa compounds, we focused on the most abundant candidates and chemically synthetized pure dn-*iso*-OPDA-Glu, dn-*iso*-OPDA-Gln and dn-*iso*-OPDA-His to use them as standard in targeted LC-MS analyses. The subsequent analyses corroborated that these dn-*iso*-OPDA-aa conjugates significantly accumulate in Tak-1 after wounding (Fig. 2A). In contrast, Mp*fad5* mutant, impaired in dn-*iso*-OPDA biosynthesis (Soriano et al. 2022), accumulated substantially less conjugates than WT plants indicating that these compounds are primarily synthesized from the canonical dn-OPDA biosynthetic pathway (Fig. 2A). Next, we analyzed if another stress inducing dn-OPDA accumulation, such as herbivory, could also promote the accumulation of dn-*iso*-OPDA-aa conjugates. Indeed, Tak-1 plants accumulated high levels of dn-*iso*-OPDA-aa conjugates after herbivory challenge (Fig. 2B). Similar to Mp*coi1*, Mp*mycy* mutant showed only minor defects in the accumulation of these compounds, indicating that the COI1/MYC pathway is only marginally involved in their biosynthesis (Fig. 2B).

In summary, metabolic profiling of *Marchantia* Tak-1 plants led to the identification of previously unknown derivatives of dn-*iso*-OPDA, the ligand of the COI1-JAZ co-receptor. The biosynthesis of dn-*iso*-OPDA-aa conjugates depended on the canonical dn-OPDA biosynthesis pathway (Soriano et al. 2022), but was mostly unaffected by the COI1 signaling pathway.

### The accumulation of dn-OPDA-amino acid conjugates is conserved in bryophytes and lycophytes

In addition to *Marchantia*, accumulation of dn-*iso*-OPDA has been recently reported in several bryophytes and lycophytes (Monte et al. 2022; Chini et al. 2023). To evaluate if dn-*iso*-OPDA-aa conjugates are synthetized in these plants, the accumulation of these compounds in response to wounding was studied in different lycophytes and bryophytes, as well as plants unable to accumulate dn-*iso*-OPDA, such as the angiosperm *A. thaliana* and the charophyte *K. nitens*.

**Figure 3.**
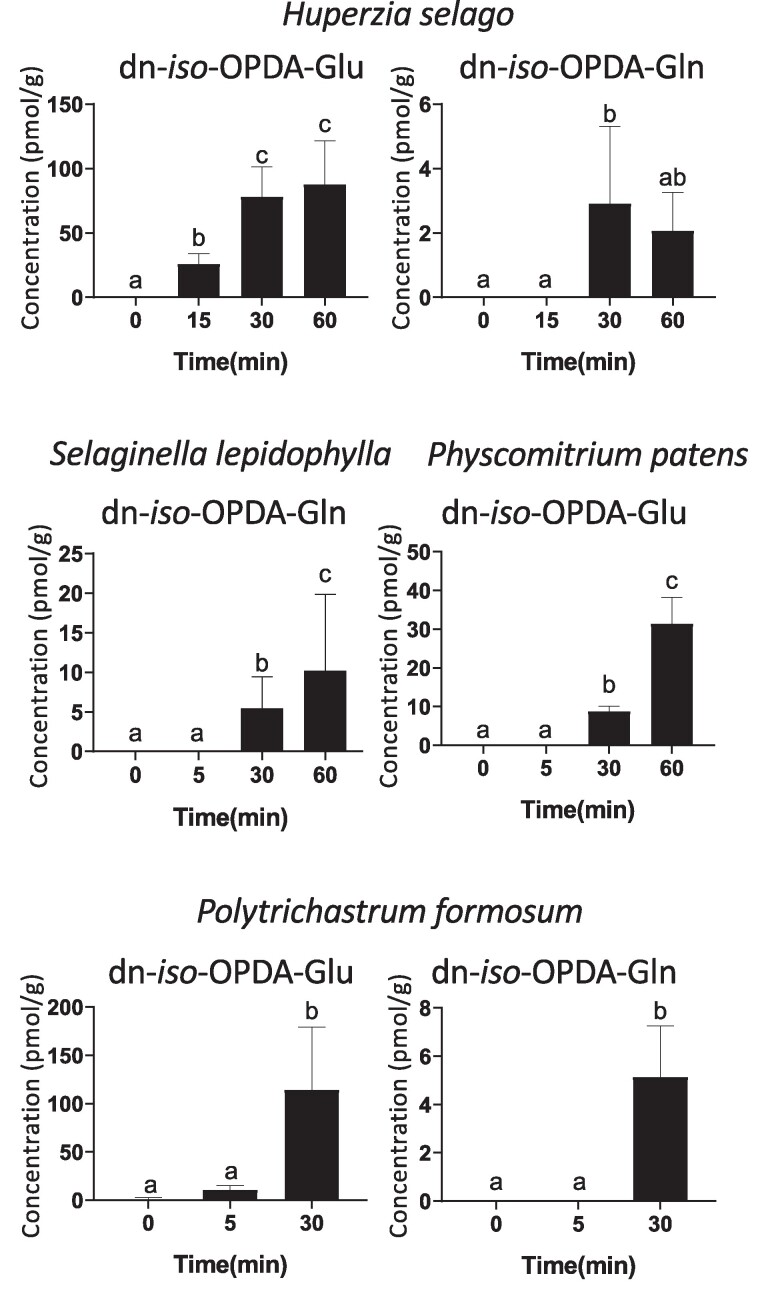
Accumulation of dn-*iso*-OPDA-aa conjugates in representative lycophytes and bryophytes. Time-course accumulation of dn-*iso*-OPDA conjugates [pmol/fresh weight (g)] in lycophyte (*S. lepidophylla* and *H. selago*) and bryophyte (*P. patens* and *P. formosum*) plants (*N* = 5–20). Plants were wounded and damaged tissues were collected after the indicated times. Unwounded plants (0) were included as control. Data shown as mean ± SD of three or four biological replicates. Experiments were repeated twice with similar results. Letters indicate significant different samples according to the one-way ANOVA/Tukey HSD post hoc test (*P* < 0.05).

As expected, in Arabidopsis and Klebsormidium, which lack dn-*iso*-OPDA, the corresponding dn-*iso*-OPDA-aa conjugates were not detected. In contrast, wounding induced the accumulation of dn-*iso*-OPDA-Glu and -Gln in the lycophyte *Huperzia Selago* and the bryophyte *Polytrichastrum formosum* (Fig. 3), whereas the lycophyte *Selaginella lepidophylla* and the bryophytes *Physcomitrium patens* respectively accumulated dn-*iso*-OPDA-Glu and -Gln in response to wounding (Fig. 3). dn-*iso*-OPDA-His was not detected in any of the analyzed plants. Although the levels of and the ratio among dn-*iso*-OPDA-aa conjugates may vary in different plants, dn-*iso*-OPDA-Glu was the most accumulated among these compounds in all plants analyzed with the only exception of *S. lepidophylla*.

Altogether, these data show that dn-OPDA-aa conjugates are not specifically occurring in *Marchantia* but they are evolutionarily conserved stress-inducible compounds in plants that synthesize dn-*iso*-OPDA as the bioactive hormone, including bryophytes and lycophytes.

### Role of MpGH3A in the biosynthesis of dn-OPDA-amino acid conjugates

Conjugation of several phytohormones with amino acids have been reported (Jez 2022). In the case of JAs, auxins and SA, the enzymes involved in amino acid-conjugation belong to the GH3 family. Therefore, we reasoned that a GH3 enzyme may also conjugate dn-*iso*-OPDA with amino acids in *Marchantia*. The *Marchantia* genome contains two candidate *GH3* genes, Mp*GH3A* (Mp*6g07600*) and Mp*GH3B* (Mp*2g14010*). Mp*GH3A* was induced by OPDA and wounding in a MYC- and COI1-dependent manner, whereas Mp*GH3B* was not induced by OPDA (Supplementary Fig. S3). The fact that *JAR1*, encoding for the GH3 involved in JA conjugation in Arabidopsis, is transcriptionally induced by JA (Staswick and Tiryaki 2004), pointed Mp*GH3A* as the most likely candidate to encode the enzyme mediating dn-OPDA conjugation with amino acids. To test this hypothesis, we generate loss-of-function mutants of Mp*GH3A* by CRISPR-Cas9.

**Figure 4.**
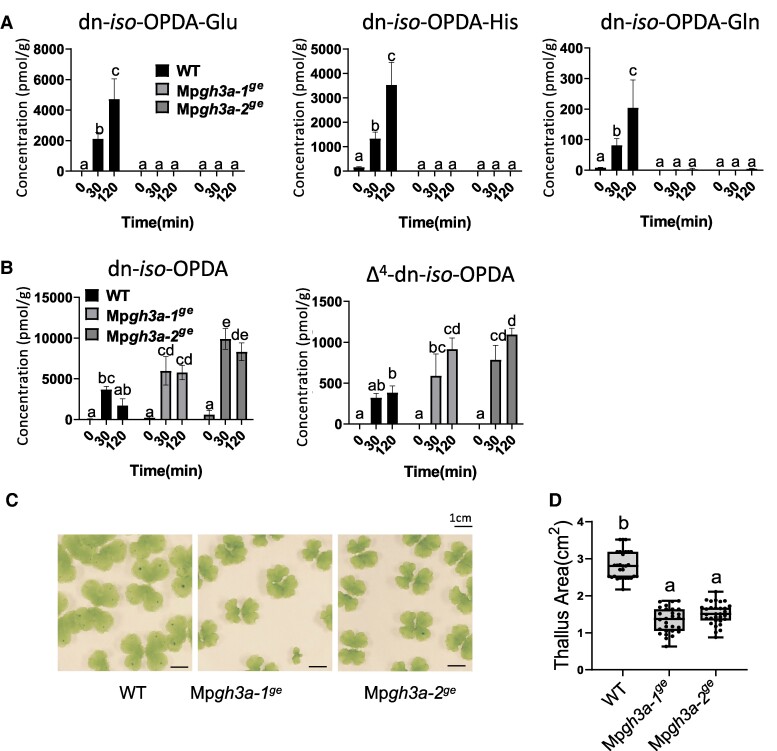
Accumulation of dn-*iso*-OPDAs in WT and Mp*gh3a^ge^* mutant plants after wounding. **A** and **B)** Quantification of dn-*iso*-OPDAs and aa-conjugates levels [pmol/fresh weight (g)] in *Marchantia* WT Tak-1 (WT) and Mp*gh3a^ge^* mutant plants after wounding. Plants were wounded, and damaged plants were collected after the indicated times. Unwounded plants (0) were included as control. Data shown as mean ± SD of three biological replicates, nine plants per each replicate. Experiments were repeated three times with similar results. Letters indicate significant different samples according to the one-way ANOVA/Tukey HSD post hoc test (*P* < 0.05). **C)** Images of *Marchantia* WT and mutant plants grown for 21 days on GB5. Scale bars, 1 cm. **D)** Quantification of thallus area (cm^2^) of plants (*n* = 30) shown in **C)**. The experiment was repeated three times with similar results. Box plots representation of thallus area; horizontal lines are medians, boxes show the upper and lower quartiles, and whiskers show the full data range. Letters indicate significant different samples according to the one-way ANOVA/Tukey HSD post hoc test (*P* < 0.05).

First, two alleles were confirmed as genuine loss-of-function mutants. Mp*gh3a-1^ge^* carried a deletion of 569 nucleotides causing a substantial loss of the first exon and a premature stop codon (Supplementary Fig. S4A). The second allele Mp*gh3a-2^ge^* carried a deletion of 911 nucleotides, resulting in the loss of most of the second and third exons and a premature stop codon (Supplementary Fig. S4A); therefore, both alleles are predicted to be complete loss-of-function mutations. Next, the accumulation of dn-*iso*-OPDA-aa conjugates was analyzed in Mp*gh3a-1^ge^* and Mp*gh3a-2^ge^* plants after wounding. Mp*gh3a^ge^* mutants were unable to accumulate dn-*iso*-OPDA-aa conjugates that were significantly induced by wounding in wild-type (WT) plants (Fig. 4A). The only exception was the detection of traces of dn-*iso*-OPDA-Gln in Mp*gh3a^ge^* plants, although at significantly lower levels in wounded Mp*gh3a^ge^* plants compared with mock Tak-1 plants. In addition, dn-*cis*-OPDA and the bioactive dn-*iso*-OPDA and Δ^4^-dn-*iso*-OPDA accumulated more in Mp*gh3a^ge^* mutants compared with Tak-1 plants after wounding (Fig. 4B and Supplementary Fig. S4B). In addition, Mp*gh3a^ge^* plants were significantly smaller than WT (Fig. 4C and D).

In summary, these results showed that MpGH3A is required in planta for the conjugation of dn-*iso*-OPDA with different amino acids.

### Biological role of dn-OPDA-amino acid conjugates in *M. polymorpha*

In Arabidopsis, Ile conjugation with JA by AtGH3.11/JAR1 and AtGH3.10 produces the bioactive JA-Ile (Staswick and Tiryaki 2004; Delfin et al. 2022). In contrast, conjugation of auxin and SA with different amino acids generate inactivate molecules. Therefore, amino acids conjugation can either activate or deactivate different signaling molecules.

**Figure 5.**
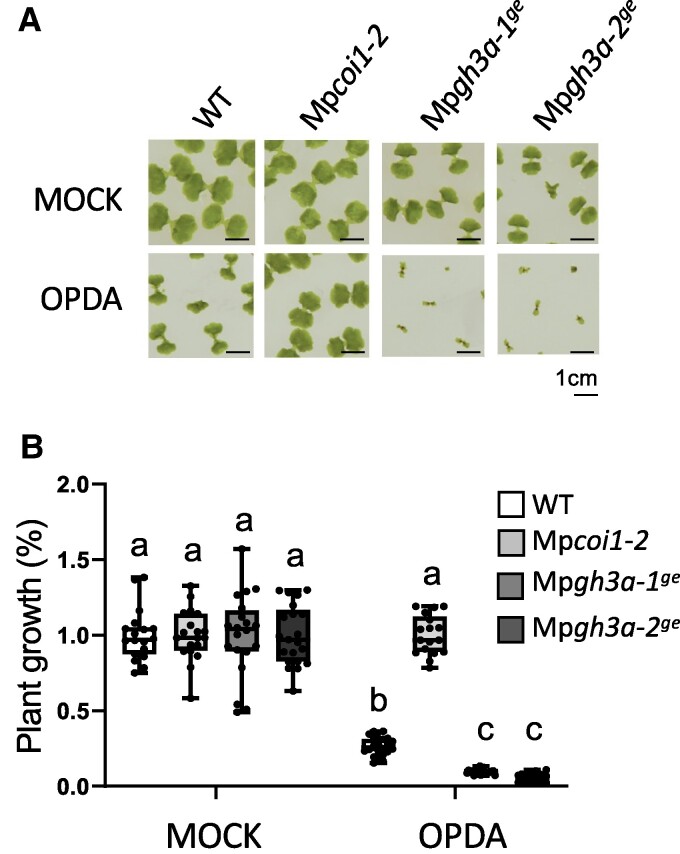
Effect of OPDA on the growth of WT and Mp*gh3a^ge^* mutant plants. **A)** Images of the plants grown for 13 days in mock plates or in the presence of the dn-OPDA precursor OPDA. **B)** Quantification of the growth inhibition induced by OPDA in wild-type Tak-1 (WT), Mp*coi1-2* and Mp*gh3a^ge^* mutant plants grown for 13 days in absence (mock) and presence of 5 μM OPDA. Data are shown as mean ± SD of three biological replicates, 20 plants per each replicate. Experiments were repeated three times with similar results. Box plots representation of growth inhibition; horizontal lines are medians, boxes show the upper and lower quartiles, and whiskers show the full data range. Letters indicate significant different samples according to the one-way ANOVA/Tukey HSD post hoc test (*P* < 0.05).

**Figure 6.**
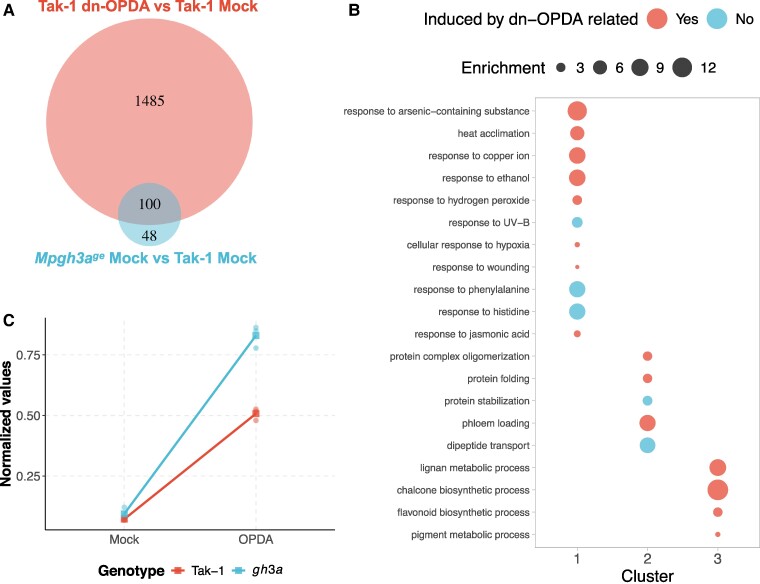
Transcriptomic analyses of *Mpgh3a-1^ge^*. **A)** Venn diagram showing DEGs upregulated after dn-OPDA treatment in Tak-1 (red) and DEGs upregulated in *Mpgh3a-1^ge^* compared with Tak-1 in Mock conditions (blue). **B)** Top 20 GO terms representing upregulated DEGs in *Mpgh3a-1^ge^* compared with Tak-1 in Mock conditions ordered according to the score integrating the *P*-value and the semantic algorithm. GO terms induced by OPDA treatment in WT plants are highlighted in red. The three GO clusters are shown in Supplementary Fig. S6. **C)** Normalized expression of 117 genes (turquoise module) from the genotype:treatment interaction effect generated by WGCNA. Orange squares represent gene expression in Tak-1 WT, while blue squares represent gene expression in *Mpgh3a-1^ge^*.

To study the biological activity of the dn-*iso*-OPDA-aa conjugates in *Marchantia*, we analyzed OPDA-regulated processes in Mp*gh3a^ge^* plants. First, OPDA sensitivity of Mp*gh3a^ge^* mutants was tested; OPDA induced significantly stronger growth inhibition in Mp*gh3a^ge^* mutants compared with Tak-1 plants (Fig. 5A), suggesting that Mp*gh3a^ge^* plants response to OPDA is enhanced compared with WT. Next, analyses of dn-OPDA-specific marker genes showed that, the Mp*gh3a^ge^* plants showed significantly higher expression of Mp*DIR* and Mp*HBLH4* compared with the WT plants upon wounding (Supplementary Fig. S5). Finally, to evaluate the impact of the upregulation of dn-OPDA pathway on the transcriptome of Mp*gh3a-1^ge^* plants, RNA-Seq analyses were carried out on mutants and WT plants. The principal component analysis (PCA) plot showed that replicate samples clustered together. In addition, the treatment factor showed a more significant impact in gene expression compared with the genotype factor (Supplementary Fig. S6A). In basal conditions, 256 genes were differentially expressed genes (DEGs) in Mp*gh3a^ge^* compared with WT plants (148 up- and 108 downregulated, Supplementary Table S1). Most of the upregulated DEGs in Mp*gh3a-1^ge^* in basal conditions were induced by OPDA treatment in WT plants (Fig. 6A), confirming a partial activation of the dn-OPDA pathway in the mutant. Gene ontology (GO) enrichment analysis showed that approximately half of the GO terms upregulated in basal conditions in the Mp*gh3a-1^ge^* mutant are also upregulated in response to OPDA treatment in WT plants (Supplementary Fig. S6B and Table S2). Furthermore, biological terms such as “response to JA,” “response to wounding” or response to additional stresses regulated by dn-OPDA were significantly upregulated in Mp*gh3a-1^ge^* in basal conditions (Fig. 6B and Supplementary Fig. S6C). In response to OPDA, 142 genes showed a genotype:treatment interaction effect, responding to dn-OPDA in Mp*gh3a-1^ge^* differently to WT plants response (Supplementary Table S1). Most of these genes were upregulated by OPDA in WT plants (Supplementary Fig. S7A). In addition, weighted gene co-expression analysis (WGCNA) also defined different co-expressed modules; the vast majority of the genes (117) showed a steeper induction by OPDA in the mutant compared with WT (turquoise module; Fig. 6C and Supplementary Fig. S7B). In summary, the transcriptomic analyses showed that the absence of the Mp*GH3A* gene enhances dn-OPDA-related gene expression both in basal conditions and after dn-OPDA treatment.

**Figure 7.**
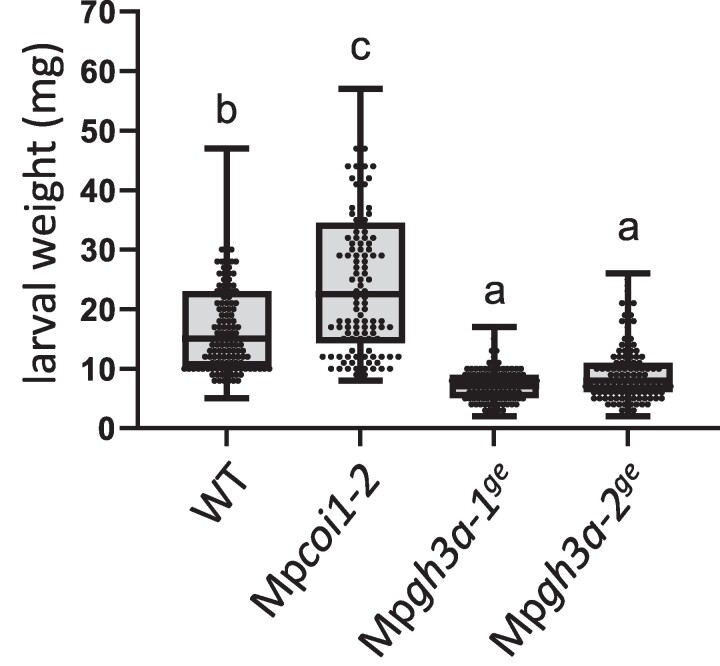
Mp*gh3a^ge^* mutants show enhanced resistance against *S. exigua*. *S. exigua* larval weight after 7 days feeding on *Marchantia* thalli of wild-type Tak-1 (WT), Mp*coi1-2* and Mp*gh3a^ge^* mutants (*n* = 114 to 136). Box plots representation of larval weight (mg); horizontal lines are medians, boxes show the upper and lower quartiles, and whiskers show the full data range. Different letters indicate significant differences evaluated by one-way ANOVA/Tukey HSD post hoc test (*P* < 0.05). Experiments were repeated four times with similar results.

Since the dn-OPDA pathway regulates biotic stress responses in *Marchantia*, including defenses against the generalist herbivore Spodoptera (Monte et al. 2018; Peñuelas et al. 2019), we challenged Tak-1, Mp*gh3a^ge^*, and Mp*coi1-2* plants with larvae of this insect. As previously reported, Mp*coi1-2* mutants were more susceptible than WT plants to herbivory. In contrast, Mp*gh3a^ge^* mutants were significantly more resistant to *Spodoptera exigua* than WT plants (Fig. 7).

Altogether, our results show that Mp*gh3a^ge^* mutants have an enhanced accumulation of dn-*iso*-OPDA, compared with WT, due to the lack of amino acid conjugation. As a consequence, dn-*iso-*OPDA responses are significantly enhanced in the mutant than in WT plants. Therefore, these results show that conjugation with amino acids inactivates dn-*iso*-OPDA hormonal bioactivity in *M. polymorpha*.

## Discussion

Endogenous plant hormones, including JAs, are tightly regulated, since they modulate the vast majority of plant responses and developmental processes (Blázquez et al. 2020; Gasperini and Howe 2024). Complementary to the biosynthetic pathway, hormone catabolism is a key process regulating the dynamic regulation of hormone homeostasis. For example, auxin homeostasis is regulated by several modifications of the bioactive IAA, including oxidation, methylation, and conjugation (Stepanova and Alonso 2016; Abbas et al. 2018). It is well-described that GH3 enzymes catalyze the conjugation of amino acids with IAA, which in turn restrain its activity (LeClere et al. 2002; Rampey et al. 2004; Westfall et al. 2012; Jez 2022). In contrast, Ile conjugation to JA produces the bioactive JA-Ile in tracheophyte plants, exemplifying that similar metabolic processes may have opposite biological effects (Staswick and Tiryaki 2004; Chini et al. 2023).

Multiple processes have been reported to regulate JAs activity in angiosperms, including hydroxylation, carboxylation, decarboxylation, methylation, sulfation, and O-glycosylation (Wasternack and Feussner 2018; Li et al. 2021; Yi et al. 2024). However, in *Marchantia*, and bryophytes in general, catabolic processes regulating the bioactive dn-OPDA homeostasis have not been reported to date.

Here, we describe an untargeted metabolomic analyses carried out in the bryophyte model *M. polymorpha* in response to wounding. In addition to compounds belonging to the canonical chemical families regulated by JAs in several plant species, such as oxylipins, terpenoids, phenylpropanoids, and flavonoids, a previously unreported family of compounds was identified (Fig. 1). The synthesis of chemical standards and targeted LC-MS analyses confirmed these compounds as amino acid conjugates of dn-OPDA (Fig. 2). To evaluate if conjugation of the bioactive dn-*iso*-OPDA regulates its activity, we first generated loss of function mutants of *MpGH3A* and showed that this enzyme is responsible for the synthesis of the conjugates (Fig. 4). Besides the lack of dn-OPDA-aa conjugates, Mp*gh3a^ge^* mutants also showed a constitutive accumulation of the bioactive dn-OPDAs as well as activation of the dn-OPDA transcriptional cascade (Figs. 4 and 6). Mp*gh3a^ge^* plants showed OPDA hypersensitivity and enhanced activation of defenses regulated by dn-OPDA (Figs. 5 and 7). Therefore, the analysis of Mp*gh3a^ge^* mutants suggested that dn-*iso*-OPDA-aa conjugates are inactive metabolites regulating the homeostasis of the bioactive dn-*iso*-OPDA.

In angiosperm plants, different set of amino acid conjugates to IAA have been reported as storage of the bioactive IAA, via regulated hydrolase, or irreversible inactive compounds (Rampey et al. 2004). The peptidases of the M20D family, including IAA-LEUCINE RESISTANT1 and its homologs, mediate the hydrolysis of auxin conjugates (Bartel and Fink 1995; LeClere et al. 2002). In contrast to auxin, SA conjugation to aspartic acid is irreversible since the SA-Asp conjugate cannot be converted into free SA (Chen et al. 2013). To date, the possibility that dn-*iso*-OPDA-aa conjugation is a reversible process is still unaddressed and future research will determine if amino acid conjugation is a regulated, reversible process. At this moment, we cannot rule out that, similar to IAA-aa conjugates (Kowalczyk and Sandberg 2001), the most abundant dn-*iso*-OPDA-aa conjugates identified here may be irreversible inactive compounds, whereas less abundant dn-*iso*-OPDA-aa conjugates may be hydrolyzed and their dynamic regulation may make it difficult to identify them in planta. Alternatively, similar to OPDA-aa conjugates, only a subgroup of dn-*iso*-OPDA-aa conjugates may undergo hydroxylation, independently on their accumulation levels (Široká et al. 2022; Brunoni et al. 2023). In this context, only one *Marchantia* homolog of angiosperm amidohydrolase was identified and in vitro analyses showed that MpILR can accept different auxin conjugates as substrates (Campanella et al. 2018; Bowman et al. 2021; Široká et al. 2022). However, the naturally occurring IAA-Gly and IAA-Val were not among the MpILR substrates in vitro (Campanella et al. 2018). In addition, the amount of auxin conjugates in bryophytes is significantly lower than in angiosperms (Sztein et al. 1999; Záveská Drábková et al. 2015; Bowman et al. 2021). Indeed, under basal conditions, the levels of IAA conjugates were below our detection limits, similar to previous results (Kaneko et al. 2020), arguing against a role of MpILR in regulating auxin homeostasis. Therefore, to evaluate the hypothetical reversibility of inactive dn-*iso*-OPDA-aa conjugates to bioactive dn-*iso*-OPDA, it will be interesting to study the role of MpILR on dn-*iso*-OPDA-aa conjugates.

In contrast to Mp*GH3A*, Mp*GH3B* was not transcriptionally regulated by dn-OPDA (Monte et al. 2018; Peñuelas et al. 2019). However, the apparent lack of regulation of Mp*GH3B* by dn-OPDA does not rule out that MpGH3B may be involved in regulating the homeostasis of dn-*iso*-OPDA. For example, At*GH3.10* is weakly induced by JA, but AtGH3.10 enzyme could conjugate JA to several amino acids, showing a redundant role of AtGH3.10 and AtJAR1 in the biosynthesis of the bioactive JA-Ile in Arabidopsis (Delfin et al. 2022). Furthermore, although Mp*GH3A* expression is upregulated by dn-OPDA, the mutants in the perception and the signaling pathway of dn-OPDA, Mp*coi1* and Mp*mycy*, are only slightly affected in biosynthesis of dn-OPDA-aa conjugates (Figs. 1 and 2), supporting a minor role of the transcriptional feedback loop in dn-OPDAs biosynthesis in *Marchantia*. These data also suggest that the basal level of MpGH3A enzyme is sufficient to promote the majority of the conjugation of dn-OPDA with amino acids. In this context, Mp*gh3a^ge^* mutant plants accumulated residual traces of dn-*iso*-OPDA-Gln after wounding (Fig. 4), suggesting a minor redundancy in the dn-OPDA conjugation activity. Therefore, further research is required to define a putative role of MpGH3B in dn-OPDA homeostasis.

Furthermore, in the bryophyte and lycophyte plants analyzed here, dn-*iso*-OPDA-Glu was the most abundant dn-*iso*-OPDA conjugate in most cases (Fig. 3), whereas dn-*iso*-OPDA-His was only identified in *M. polymorpha*. These results may suggest a species specificity in the biosynthesis of some of these compounds; however, due to the deactivation role of dn-*iso*-OPDA conjugates, it seems unlikely that different dn-*iso*-OPDA conjugates may hold specific functions.

This work describes dn-*iso*-OPDA conjugation as a process that inactivates the bioactive JA molecule in *Marchantia*. However, additional processes regulating JA homeostasis in angiosperms have been described, including hydroxylation, carboxylation, oxidation and sulfation among others (Wasternack and Feussner 2018; Li et al. 2021). It will be interesting to evaluate if any additional JA inactivation processes may be conserved in bryophytes. Noteworthy, in Arabidopsis different catabolic auxin processes, such as conjugation and oxidation, were described to be coordinately regulated to achieve the most beneficial auxin homeostasis (Bowman et al. 2021). Therefore, it would be interesting to evaluate if additional dn-OPDA inactivation processes may interplay with dn-*iso*-OPDA-aa conjugation to obtain the best JA homeostasis in bryophytes.

In summary, our results show that the signaling activity of dn-*iso*-OPDA is reduced by its conjugation with different amino acids. These results illustrate a dichotomous role of JA conjugation in land plants: JA conjugation to Ile produces the bioactive JA-Ile in tracheophyte plants, whereas conjugation of dn-*iso*-OPDA to amino acids deactivates the bioactive dn-*iso*-OPDA, bioactive ligand of COI1-JAZ co-receptor, in bryophyte and lycophyte plants.

## Materials and methods

### Plant material

*Marchantia polymorpha* accession Tak-1 was used as the WT. In this genetic background, we used CRISPR-Cas9 nickase-mediated mutagenesis with Mp*GH3A* as the target. Four different pairs of gRNAs were designed in the first exon of the gene (Supplementary Table S3). The eight gRNAs were then first cloned into pBC-GE12, pBC-GE23, pBC-GE34, and pMPGE_EN04 vectors. Next, they were transferred by LR reaction into the pMpGE017 binary vector carrying the CRISPRCas9 nickase. WT plants were transformed and thalli were selected by hygromycin resistance employing the regenerating thalli transformation (Kubota et al. 2013). To select mutant plants, genomic DNA of hygromycin-resistant explants was extracted and sequenced as previously described (Kneeshaw et al. 2022; Soriano et al. 2022). In addition, the Mp*coi1-2* and Mp*mycy* mutants were used as control in some experiments (Monte et al. 2018; Peñuelas et al. 2019).

### Chemicals

*Cis*-12-oxo-phytodienoic acid (OPDA) and dinor-12-oxophytodienoic acid (dn-OPDA) were purchased from Cayman Chemical Co. (Ann Arbor, MI, USA), whereas dn-*cis*-OPDA and dn-*iso*-OPDA were previously synthesized (Chini et al. 2023).

### Culture conditions and wounding

Plants were routinely grown at 21 °C, under continuous white light (50–60 mmol m^−2^ s^−1^) on half-strength Gamborg's B5 medium Petri plates containing 1% agar. For JA quantification, plants were grown for 2 weeks. For wounding assays, thalli of the WT and mutant plants were mechanically wounded with tweezers, pressing all over the thallus surface (Kneeshaw et al. 2022; Soriano et al. 2022). Wounded thalli were harvested and immediately frozen in liquid nitrogen after the described times.

### Growth inhibition assays

Growth inhibition assays were performed as previously described (Kneeshaw et al. 2022; Soriano et al. 2022). Briefly, OPDA was added to the GB5 media at a 5 μM concentration. After 2 weeks, pictures of the plants were acquired with a NIKON D1-x digital camera and the area of each plant was estimated with the ImageJ software. The growth percentage was calculated as the ratio of the area of hormone-treated versus untreated plants. The assays were repeated at least three times with similar results.

### Extraction of polar and semi-polar specialized metabolites

Polar and semi-polar metabolite fraction was extracted as previously described (Routaboul et al. 2006), with some modifications. Samples consisted of 5 mg of *M. polymorpha* grinded tissues. Briefly, 1 mL of methanol/water (80/20) + 0.05% of formic acid were added to each sample. The mixtures were sonicated using an ice-cooled ultrasonication (Fischer Scientific FB15050) for 1 min, then shaken in 2-mL tubes using a ThermoMixer C (Eppendorf) at 1,400 rpm for 30 min and 4 °C. The tubes were centrifuged at 10,000 rpm for 10 min at 4 °C; the supernatants were collected in glass tubes. Next, 1 mL of extraction solution was added to the 2 mL tube containing the pellet, and the extraction procedure was repeated. The two supernatants were pooled and dried using a vacuum concentrator (SpeedVac). The dried extracts were resuspended in 100 µL Acetonitrile:Water (1:9) of ULC/MS grade. The resuspended extracts were filtered with a glass microfiber filters (Cat. NO. 1820-037, Whatman International Ltd., UK) and distributed in HPLC vials. A quality control (QC) made up of 5 µL from each sample was prepared in a separate HPLC vials.

### UPLC-MS/MS analysis of polar and semi-polar specialized metabolites and UPLC-MS/MS data processing

Extracted samples were analyzed as previously described (Boutet et al. 2022). The untargeted metabolomic data were acquired using a UHPLC system (Ultimate 3000 Thermo) coupled to quadrupole time of flight mass spectrometer (Q-TOF Impact II Bruker Daltonics, Bremen, Germany). A Nucleoshell RP 18 plus reversed-phase column (2 × 100 mm, 2.7 µm; Macherey–Nagel) was used for chromatographic separation with the mobile phases consisting in: (A) 0.1% formic acid in water; and (B) 0.1% formic acid in acetonitrile. The flow rate was 400 µL min^−1^, and the following gradient was used: 95% (A) for 1 min, followed by a linear gradient from 95% (A) to 80% (A) from 1 to 3 min, then a linear gradient from 80% (A) to 75% (A) from 3 to 8 min, a linear gradient from 75% (A) to 40% (A) from 8 to 20 min; then, 0% of (A) was held until 24 min, followed by a linear gradient from 0% (A) to 95% (A) from 24 to 27 min. Finally, the column was washed by 30% (A) at for 3.5 min then re-equilibrated for 3.5 min (35 min total run time). Mass spectrometer data were obtained with data-dependent acquisition method in both positive and negative electrospray ionization (ESI) modes using the following parameters: capillary voltage, 4.5 kV; nebulizer gas ﬂow, 2.1 bar; dry gas ﬂow, 6 L min^−1^; drying gas in the heated electrospray source temperature, 200 °C. Samples were analyzed at 8 Hz with a mass range of 100 to 1500 m/z. Stepping acquisition parameters were created to improve the fragmentation profile with a collision RF from 200 to 700 Vpp, a transfer time from 20 to 70 µs, and collision energy from 20 to 40 eV. Each cycle included a MS fullscan and 5 MS/MS CID on the 5 main ions of the previous MS spectrum.

The UPLC-MS/MS data processing pipeline was performed as in Boutet et al. (2022) with modifications. Briefly, .d data files (Bruker Daltonics, Bremen, Germany) were converted to.mzXML format using the MSConvert software (ProteoWizard package 3.0). mzXML data processing, mass detection, chromatogram building, deconvolution, samples alignment and data export were performed using MZmine 2 software (http://mzmine.github.io/) for both positive and negative data files. The ADAP chromatogram builder method was used with a minimum group size of scan 3, a group intensity threshold of 1,000, a minimum highest intensity of 1,000 and m/z tolerance of 10 ppm. Deconvolution was performed with the ADAP wavelets algorithm using the following setting: S/N threshold 8, peak duration range = 0.01 to 2 min RT wavelet range 0.01 to 0.2 min, MS2 scan were paired using a m/z tolerance range of 0.05 Da and RT tolerance of 0.2 min. Then, isotopic peak grouper algorithm was used with a m/z tolerance of 10 ppm and RT tolerance of 0.1. All the peaks were filtered using feature list row filter keeping only peaks with MS2 scan. The alignment of samples was performed using the join aligner with an m/z tolerance of 10 ppm, a weight for m/z and RT at 1, a retention time tolerance of 0.2 min. Metabolites accumulation was normalized according to the weight of seeds used for the extraction. Pooled QC sample injections across LC-MS/MS run were used to evaluate the quality of the run and untargeted metabolomic dataset. The relative standard deviation across the QC samples was calculated for each metabolic feature detected. The metabolic features were filtered according to their QC variation and only metabolic features with QC variation < 25% were kept. The metabolomic data and metadata have been deposited at the MassiVE data repository portal with the identifier MSV000096228 (doi: 10.25345/C53J39C74).

### Molecular network and annotation of untargeted metabolomic data

As described previously, molecular networks were generated with MetGem software 20 (https://metgem.github.io) using the.mgf and.csv files obtained with MZmine2 analysis. The molecular network was optimized for the ESI+ and ESI− datasets, and different cosine similarity score thresholds were tested. ESI− and ESI+ molecular networks were both generated using a cosine score threshold of 0.7 Molecular networks were exported to Cytoscape software (https://cytoscape.org/) to format the metabolic categories.

Metabolite annotation was performed in 4 consecutive steps. First, the “custom database search” module from Mzmine was used to compare the obtained LC-MS/MS data with the IJPB chemistry and metabolomic platform homemade experimental (m/z absolute tolerance of 0.005 and RT tolerance of 0.2 min) and exact mass (m/z absolute tolerance of 0.005 Da or 10 ppm) libraries containing respectively 166 standards or experimental common features (RT, m/z) and 1,112 ion known m/z. Second, the ESI− and ES+ metabolomic data used for molecular network analyses were searched against the available MS2 spectral libraries (Massbank NA, GNPS Public Spectral Library, NIST14 Tandem, NIH Natural Product and MS-Dial), with absolute m/z tolerance of 0.02, 4 minimum matched peaks and minimal cosine score of 0.8 (Fig. 3A). Third, not-annotated metabolites that belong to molecular network clusters containing annotated metabolites from steps 1 and 2 were assigned to the same metabolic category. Following this annotation approach, 20% of the metabolic features of the LC-MS/MS dataset were assigned to one of the metabolic categories identified. Fourth, the putative annotations of key metabolic features that were highlighted by statistical analyses was verified using Sirius, software or by checking the M2 spectra.

### Metabolites table and redundancy in MS

Redundancy cleaning due to the MS technique was performed using a local application (see Code availability) built using the freely available Shiny R package (https://cran.r-project.org/web/packages/shiny/index.html). It allows to identify the formation of adducts complementary to the protonated form in positive mode as: sodium potassium and ammonium and de-protonated in negative mode as: sodium, potassium, chlorine, formic acid, nitric acid, acetate, formic acid clusters associated with sodium by comparing the masses and retention time.

In the same way, the complementarity of positive and negative modes for certain metabolites was also eliminated by cross-referencing the modes of these complementary forms with those of their adducted forms. Both analyses were done using a mass tolerance parameter of 0.006 Da and a retention time of 0.2 min.

### Statistical analyses of metabolomic data

Statistical analyses were performed using Metaboanalyst 5.0 software (Pang et al. 2021). In particular, an ANOVA has been conducted to identify differentially accumulated metabolites among control and stress condition, and genotypes (adjusted *P* value < 0.05).

### Synthesis of dn-*iso*-OPDA amino acid conjugates

#### Synthesis of dinor-iso-OPDA-Glu

To a solution of dinor-*iso*-OPDA (Wang et al. 2021) (10.8 mg, 41 µmol, 1 eq.) and Et_3_N (12.0 µL, 82 µmol, 2 eq.) in THF (0.40 mL) was added ethyl chloroformate (4.0 µL, 41 µmol, 1 eq.) at 0 °C. After being stirred at 0 °C for 2 h, sodium L-glutamate monohydrate (15.3 mg, 82 µmol, 2 eq.) dissolved in aq. diisopropylethylamine (45 µL, 246 µmol, 6 eq.) in H_2_O (400 µL) was added the above mixture and stirred for 2 h at room temperature. The reaction mixture was then acidified with 1 m aq. HCl and extracted with CHCl_3_. The combined organic layer was washed with brine, dried over Na_2_SO_4_, and concentrated under reduced pressure. The residue was directly purified by RP-HPLC after membrane filtration (COSMOSIL Cholester column F20 × 250 mm, Flow rate 8.0 mL/min, detection 220 nm, eluent: MeCN + 1%AcOH, B: H_2_O + 1%AcOH, 0 to 5 min 30%A, 5 to 20 min 30%to 70%A, 20 to 25 min 70%A, 25 to 26 min 70% to 100%A, 26 to 30 min 100%A; t_R_ 20.6 min) to give dinor-*iso*-OPDA-Glu (7.3 mg, 45%) as a pale yellow oil (Supplementary Fig. S8). [a]_D_^19^ −7.33 (c 0.35, MeOH); ^1^H (400 MHz, CD_3_OD) d 5.26 (dt, *J* = 10.5, 7.3 Hz, 1H), 5.09 (dt, *J* = 10.5, 7.3 Hz, 1H), 4.33 (dd, *J* = 9.4, 5.0 Hz, 1H), 2.83 (d, *J* = 6.9 Hz, 2H), 2.50 to 2.46 (m, 2H), 2.41 (t, *J* = 7.3 Hz, 2H), 2.33 to 2.23 (m, 4H), 2.17 (t, *J* = 7.3 Hz, 2H), 2.13 to 2.02 (m, 1H), 2.07 (quin, *J* = 7.3 Hz, 2H), 1.84 (sext, *J* = 7.3 Hz, 1H), 1.57 (quin, *J* = 7.3 Hz, 2H), 1.50 (quin, *J* = 7.3 Hz, 2H), 1.30 (quin, *J* = 7.3 Hz, 2H), 0.86 (t, *J* = 7.3 Hz, 3H); ^13^C (100 MHz, CD_3_OD) d 212.44, 178.07, 176.27, 176.20, 174.95, 140.01, 133.19, 126.44, 52.94, 36.53, 35.15, 32.17, 31.26, 30.25, 30.19, 28.18, 27.86, 26.61, 21.93, 21.54, 14.54: HRMS (ESI neg.) [M-H]^−^ C_21_H_30_NO_6_^−^, calcd. 392.2079, found 392.2068.

#### Dinor-*iso*-OPDA-Gln

To a solution of dinor-*iso*-OPDA (10.8 mg, 41 µmol, 1 eq.) and Et_3_N (12.0 µL, 82 µmol, 2 eq.) in THF (0.40 mL) was added ethyl chloroformate (4.0 µL, 41 µmol, 1 eq.) at 0 °C. After being stirred at 0 °C for 2 h, L-glutamine (12.1 mg, 82 µmol, 2 eq.) dissolved in aq. diisopropylethylamine (45 µL, 246 µmol, 6 eq.) in H_2_O (400 µL) was added the above mixture and stirred for 2 h at room temperature. The reaction mixture was then acidified with 1 m aq. HCl and extracted with CHCl_3_. The combined organic layer was washed with brine, dried over Na_2_SO_4_, and concentrated under reduced pressure. The residue was directly purified by RP-HPLC after membrane filtration (COSMOSIL Cholester column F20 × 250 mm, Flow rate 8.0 mL/min, detection 220 nm, eluent: MeCN + 1%AcOH, B: H_2_O + 1%AcOH, 0 to 5 min 30%A, 5 to 20 min 30% to 70%A, 20 to 25 min 70%A, 25 to 26 min 70% to 100%A, 26 to 30 min 100%A; t_R_ 18.6 min) to give dinor-iso-OPDA-Glu (5.3 mg, 33%) as a pale yellow oil (Supplementary Fig. S8). [a]_D_^18^ −2.78 (c 0.27, MeOH); ^1^H (400 MHz, CD_3_OD) d 5.26 (dtt, *J* = 10.5, 7.3, 1.4 Hz, 1H), 5.09 (dtt, *J* = 10.5, 7.3, 1.4 Hz, 1H), 4.29 (dd, *J* = 9.2, 5.0 Hz, 1H), 2.83 (d, *J* = 7.3 Hz, 2H), 2.51 to 2.45 (m, 2H), 2.41 (t, *J* = 7.8 Hz, 2H), 2.29 to 2.13 (m, 7H), 2.06 (quin, *J* = 7.3 Hz, 2H), 1.84 (sext, *J* = 7.3 Hz, 1H), 1.57 (m, 2H), 1.50 (quin, *J* = 7.3 Hz, 2H), 1.30 (quin, *J* = 7.3 Hz, 2H), 0.89 (t, *J* = 7.3 Hz, 3H); ^13^C (100 MHz, CD_3_OD) d 212.43, 178.07, 177.67, 176.20, 174.87, 140.01, 133.20, 126.44, 53.21, 36.56, 35.15, 32.77, 32.17, 30.27, 30.19, 28.51, 28.18, 26.59, 21.93, 21.54, 14.54: HRMS (ESI neg.) [M-H]^−^ C_21_H_31_N_2_O_5_^−^, calcd. 391.2238, found 391.2233.

#### Dinor-*iso*-OPDA-His

To a solution of dinor-*iso*-OPDA (10.8 mg, 41 µmol, 1 eq.) and Et_3_N (12.0 µL, 82 µmol, 2 eq.) in THF (0.40 mL) was added ethyl chloroformate (4.0 µL, 41 µmol, 1 eq.) at 0 °C. After being stirred at 0 °C for 2 h, L-Histidine (12.8 mg, 82 µmol, 2 eq.) dissolved in aq. diisopropylethylamine (45 µL, 246 µmol, 6 eq.) in H_2_O (400 µL) was added the above mixture and stirred for 2 h at room temperature. The reaction mixture was then acidified with 1 m aq. HCl and washed with CHCl_3_. The remaining aqueous layer was concentrated under reduced pressure. The residue was directly purified by RP-HPLC after membrane filtration (COSMOSIL Cholester column F20 × 250 mm, Flow rate 8.0 mL/min, detection 220 nm, eluent: MeCN + 1%AcOH, B: H_2_O + 1%AcOH, 0 to 5 min 30%A, 5 to 20 min 30% to 50%A, 20 to 21 min 50% to 100%A, 21 to 25 min 100%A; t_R_ 14.4 min) to give dinor-iso-OPDA-His (4.8 mg, 29%) as a pale yellow oil (Supplementary Fig. S8). [a]_D_^19^ −4.81 (c 0.24, MeOH); ^1^H (400 MHz, CD_3_OD) d 8.83 (s, 1H), 7.33 (s, 1H), 5.34 (dt, *J* = 10.5, 7.3 Hz, 1H), 5.17 (dt, *J* = 10.5, 7.3 Hz, 1H), 4.77 (dd, *J* = 8.7, 5.0 Hz, 1H), 3.34 to 3.25 (m, 1H), 3.09 (dd, *J* = 15.6, 9.2 Hz, 1H), 2.91(d, *J* = 6.9 Hz, 2H), 2.56 (m, 2H), 2.48 (t, *J* = 7.3 Hz, 2H), 2.35 (m, 2H), 2.28 to 2.14 (m, 2H), 2.15 (quint, *J* = 7.3 Hz, 2H), 1.64 to 1.50 (m, 4H), 1.40 to 1.30 (m, 2H), 0.97 (t, *J* = 7.3 Hz, 3H); ^13^C (100 MHz, CD_3_OD) d 212.34, 177.88, 176.02, 173.12, 140.02, 135.09, 133.19, 131.43, 126.43, 118.38, 52.41, 36.50, 35.14, 32.11, 30.21, 30.19, 28.14 (2C), 26.53, 21.93, 21.53, 14.53: HRMS (ESI neg.) [M-H]^−^ C_22_H_30_N_3_O_4_^−^, calcd. 400.2242, found 400.2234.

### Chemical compound measurements

Measurement of the described compounds was carried out as previously described (Chini et al. 2018, 2023). Tissue from 5 to 10 plants was pooled for each biological sample and at least three independent biological replicates were performed for each treatment. Briefly, endogenous dn-OPDAs and derivative compounds were analyzed using high-performance LC electrospray-high resolution accurate MS (HPLC-ESI-HRMS) using an Orbitrap Exploris 120 detector in Full Scan and Product Ion Scan modes. The experiment was repeated twice to four times with similar results. Data are shown as mean ± SD.

### Transcriptional analyses

For gene expression analyses, 2-week-old thalli were treated with 25 µM OPDA or wounding, and collected after the indicated time. The RNA extracted with FavorPrep Plant Total RNA Mini Kit (Vienna, Austria), whereas genomic DNA was removed by DNase digestion. Finally, sample quality was evaluated in a Bioanalyzer 2100 (Agilent Technologies, Santa Clara, CA, USA). Three independent biological replicates per sample were analyzed. For reverse transcription quantitative PCR analysis, expression of the marker genes was analyzed using Mp*ACT* (*Mp6g11010*) as a reference gene. Experiments were repeated three times with similar results. Significant difference in gene expression was analyzed using the one-way ANOVA/Tukey HSD post hoc test (*P* < 0.05).

For RNA-seq analyses, the Illumina NovaSeq X Plus platform was employed (Novogene, Cambridge UK). Total RNA was extracted and cDNA was synthesized from poly-A. Raw paired-end reads were trimmed using default parameters with fastp tool (Chen et al. 2018). The original data are available at GEO: accession GSE275561. Then, sequences were mapped and quantified to the reference genome MpTak1_v6.1 with salmon (Patro et al. 2017). Differential expression analyses were performed using DESeq2 (Love et al. 2014) following their manual instruction, and final filters of *P*-value ≤ 0.05 and log2 of Fold Change ≥ 1 and ≤−1 for up- and downregulated DEGs, respectively. No fold-change filter was applied to the genotype:treatment interaction effect approach. GO terms enrichment analysis were performed using topGO (Alexa and Rahnenfuhrer 2024), performing “new” function with biological processes and “runTest” function with “weight01” algorithm and “fisher” statistic. WGCNA (Langfelder and Horvath 2008) tool was used for co-expression analysis, running the blockwiseModules function with TOMtype = “signed” and power = 16. PCA plots of the GO terms were performed using a custom modification of the “rrgo” package (Sayols 2023). Scores similarity matrixes between terms were created using the calculateSimMatrix function, with “org.At.tair.db” as database and *simRel* (“Rel”) similarity method. Scores were calculated from *P*-values obtained previously from topGO. K-means was used to cluster the data, using Silhouette method for calculating the optimal number of clusters (centers). All plots of transcriptional data were generated using ggplot2 package (Wickham 2016), except for venn diagrams that were generated using VennDiagram package (Chen and Boutros 2011). #E64B35FF and #4DBBD5FF were selected as colors, selected from “nrc” palette of ggsci package.

### Herbivory assays

Ten plants per each genotype were grown in plates for 4 weeks. Then, 12 first-instar larvae of *S. exigua* (Entocare, Wageningen, The Netherlands) were released in each plate. Five to 10 plates were used for each genotype. After 7 to 8 days of feeding, larvae were individually collected and weighed on a precision balance. The assays were repeated at least three times with similar results. The experiment was repeated four times with similar results. Data are shown as mean ± SD.

### Accession numbers

Sequence data from this article can be found in the GenBank/EMBL data libraries under accession numbers: MpJAZ/Mp6g06230, MpCOI1/Mp2g26590, MpMYCY/MpVg00340, MpGH3A/Mp6g07600 and MpGH3B/Mp2g14010.

## Supplementary Material

kiae610_Supplementary_Data

## Data Availability

The metabolomic data and metadata have been deposited at the MassiVE data repository portal with the identifier MSV000096228 (doi:10.25345/C53J39C74). For RNA-seq analyses, the original data are available at GEO: accession GSE275561.

## Supplementary data

The following materials are available in the online version of this article.

**Supplementary Figure S1.** Chemical structure of the putative dn-OPDA–amino acid conjugates.

**Supplementary Figure S2.** Accumulation of putative dn-OPDA-aa conjugates after wounding.

**Supplementary Figure S3.** Mp*GH3A* expression is induced by OPDA and wounding in a COI1- and MYC-dependent manner.

**Supplementary Figure S4.** Generation and analyses of Mp*gh3a^ge^* mutant alleles.

**Supplementary Figure S5.** Expression of dn-OPDA marker genes in Mp*gh3a^ge^* mutant plants.

**Supplementary Figure S6.** Transcriptomic analysis of *Mpgh3a-1^ge^* in basal conditions.

**Supplementary Figure S7.** GO and WGCNA analyses of *Mpgh3a-1^ge^* in response to OPDA treatment.

**Supplementary Figure S8.** Synthetic scheme of dinor-*iso*-OPDA-L-Amino acid conjugates.

**Supplementary Table S1.** List of DEGs identified in RNA-seq analyses.

**Supplementary Table S2.** List of GO terms of the DEGs identified in RNA-seq analyses.

**Supplementary Table S3.** List of primers used in this work.

## Dive Curated Terms

The following phenotypic, genotypic, and functional terms are of significance to the work described in this paper:

JAR1 Gramene: AT2G46370
JAR1 Araport: AT2G46370

NIH Gramene: AT1G06670
NIH Araport: AT1G06670

FAD5 Gramene: AT3G15850
FAD5 Araport: AT3G15850

COI1 Gramene: AT2G39940
COI1 Araport: AT2G39940

MpCOI1 Gramene: Mp2g26590
MpCOI1 Araport: Mp2g26590

MpJAZ Gramene: Mp6g06230
MpJAZ Araport: Mp6g06230

MpGH3A Gramene: Mp6g07600
MpGH3A Araport: Mp6g07600

MpFAD5 Gramene: Mp3g10660
MpFAD5 Araport: Mp3g10660

MpGH3B Gramene: Mp2g14010
MpGH3B Araport: Mp2g14010

WGCNA Gramene: weighted gene coexpression analysis
WGCNA Araport: weighted gene coexpression analysis

MpILR Gramene: Mp1g20090
MpILR Araport: Mp1g20090
