# Supplementary material for: Dinor-12-oxo-phytodienoic acid conjugation with amino acids inhibits its phytohormone bioactivity in Marchantia polymorpha
Source: Plant Physiol. 2024 Nov 8;197(1):kiae610. doi: 10.1093/plphys/kiae610 (PMC11663715; doi:10.1093/plphys/kiae610)
Supplement: kiae610_Supplementary_Data [file kiae610_supplementary_data.zip › SI_PP2024RA02380R1.pdf]

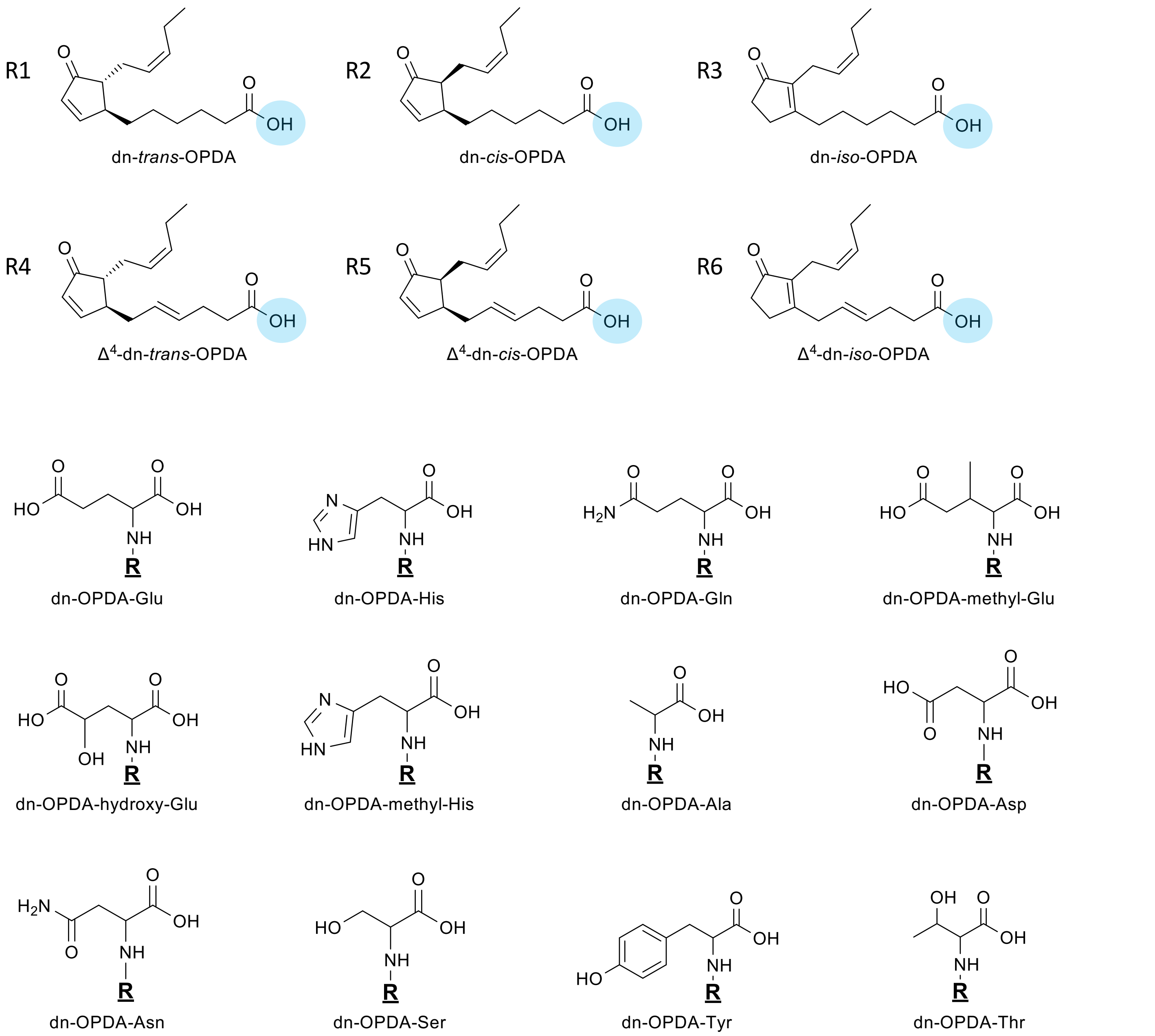

Supplementary Figure S1. Chemical structure of the putative dn-OPDA–amino acid conjugates. The structures of the putative dn-*iso*-OPDA conjugates to amino acids identified in untargeted or studied by targeted metabolomic analyses are shown. The quantification of these compounds is presented in Figure 1 and Figure S2. **R** = R1 to R6(-OH)

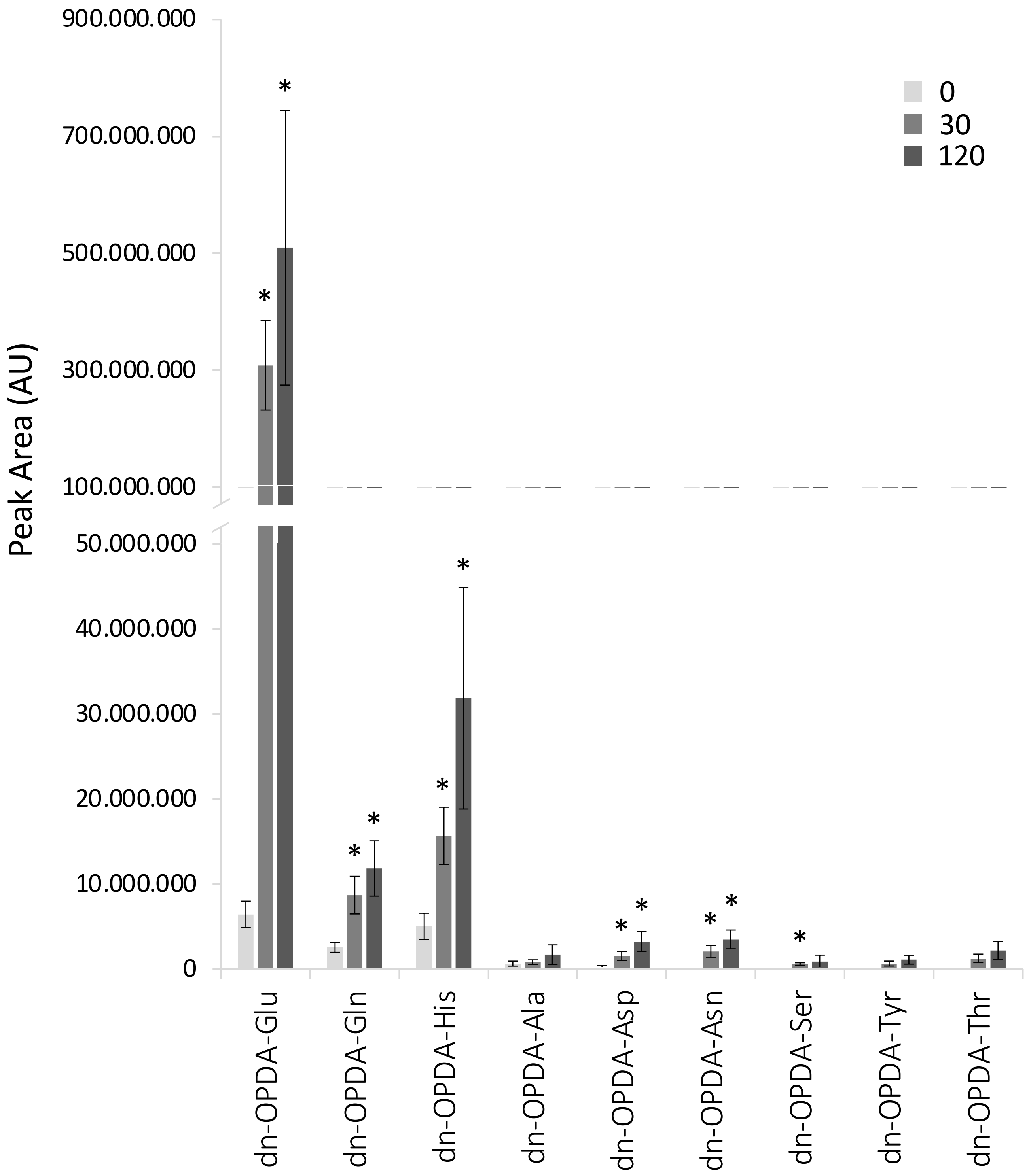

Supplementary Figure S2. Accumulation of putative dn-OPDA-aa conjugates after wounding. Accumulation of putative dn-OPDA conjugates with different amino acids in wild-type Tak-1 (WT) plants at indicated time points after wounding. Peak areas are reported in arbitrary units (AU). Untreated plants, time 0, were included as control. Data shown as mean ± s.d. of four biological replicates, 3-5 plants per each replicate. Asterisks indicate significant differences between wound-induced accumulation of control and wounded plants for each compound according to a t-test ($P < 0.05$).

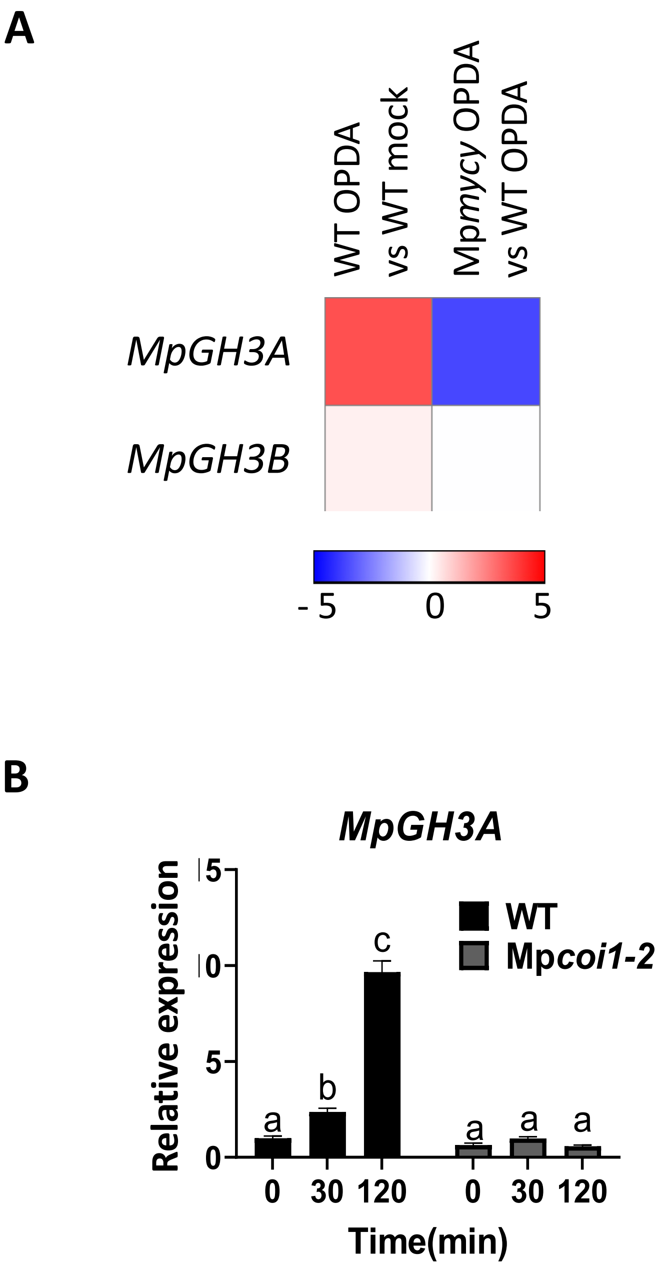

Supplementary Figure S3. Mp*GH3A* expression is induced by OPDA and wounding in a COI1- and MYC-dependent manner.

(A) Analysis of gene expression by microarray study of Mp*GH3* in mock and in response to OPDA (2 hours at 25 µM) in indicated wild-type Tak-1 (WT) and Mp*mycy-1* mutant plants (Peñuelas et al., 2019). Scale bar represent log-ratio values.

(B) RT-qPCR analysis of Mp*GH3A* expression in Tak-1 WT and Mp*coi1-2* mutant plants after wounding. Untreated plants (0) were included as control. Plant tissue was collected after the indicated time. Expression of Mp*GH3A* was normalized against Mp*ACT*. Data shown as mean ± s.d. of three biological replicates, 3-5 plants per each replicate. Experiments were repeated three times with similar results. Letters indicate significant different samples according to the one-way ANOVA/Tukey HSD post hoc test ($P < 0.05$).

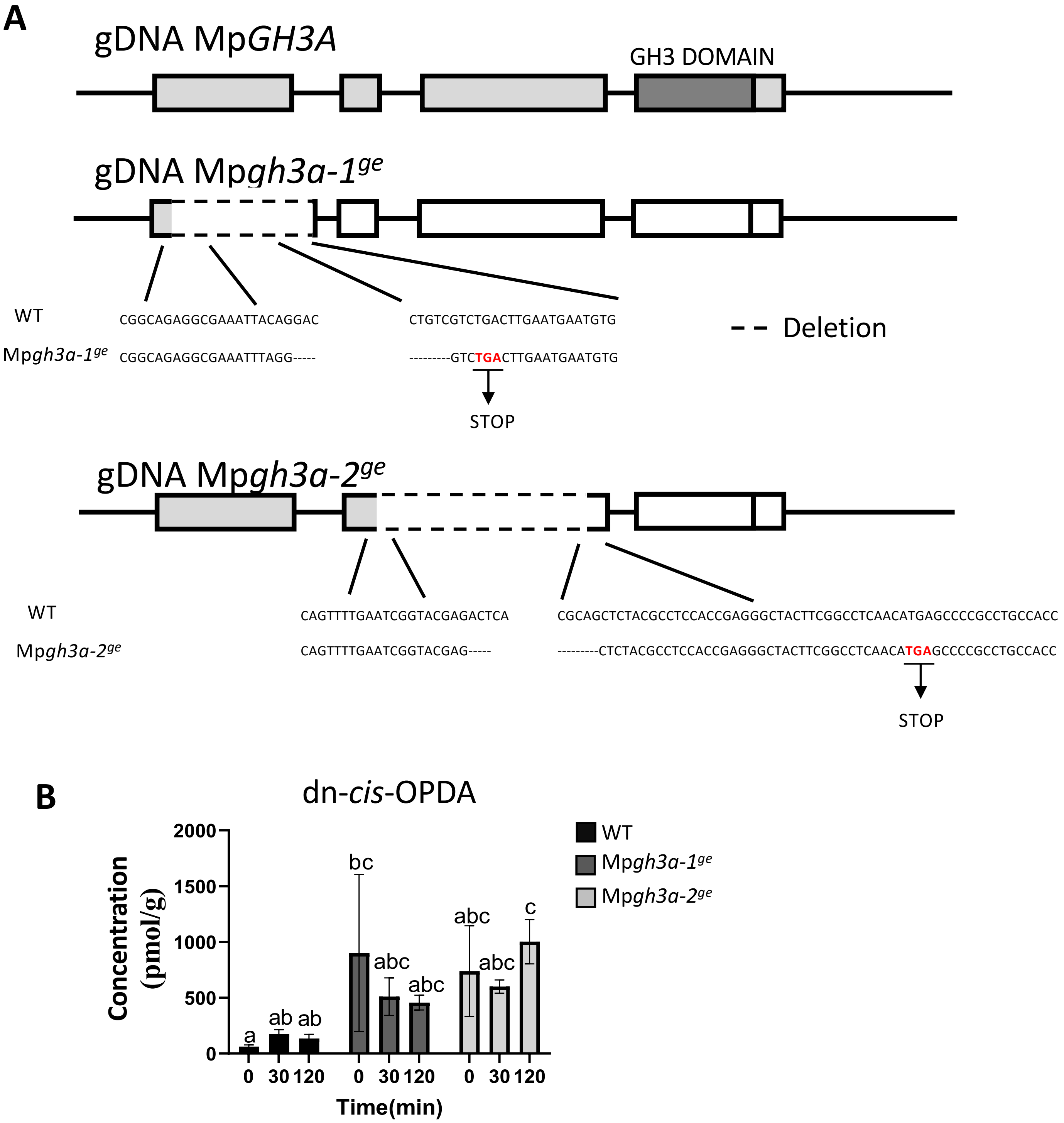

Supplementary Figure S4. Generation and analyses of Mp*gh3a*^{ge} mutant alleles.

(A) Scheme of Mp*GH3A* gene of Marchantia. Grey blocks represent exons and dark grey block highlights the sequence encoding for the GH3 domain. Dashed lines represent deletions in Mp*gh3a*^{ge} alleles; frame-shifted sequences in Mp*gh3a*^{ge} alleles causing premature stop codons (highlighted in red) are shown.

(B) Accumulation of dn-*cis*-OPDA [pmol/fresh weight (g)] in wild-type Tak-1 (WT) and Mp*gh3a*^{ge} mutant plants at indicated time points after mechanical wounding. The experiment was repeated three times with similar results. Data shown as mean ± s.d. of four biological replicates, 3-5 plants per each replicate. Letters indicate significant different samples according to the one-way ANOVA/Tukey HSD post hoc test ($P < 0.05$).

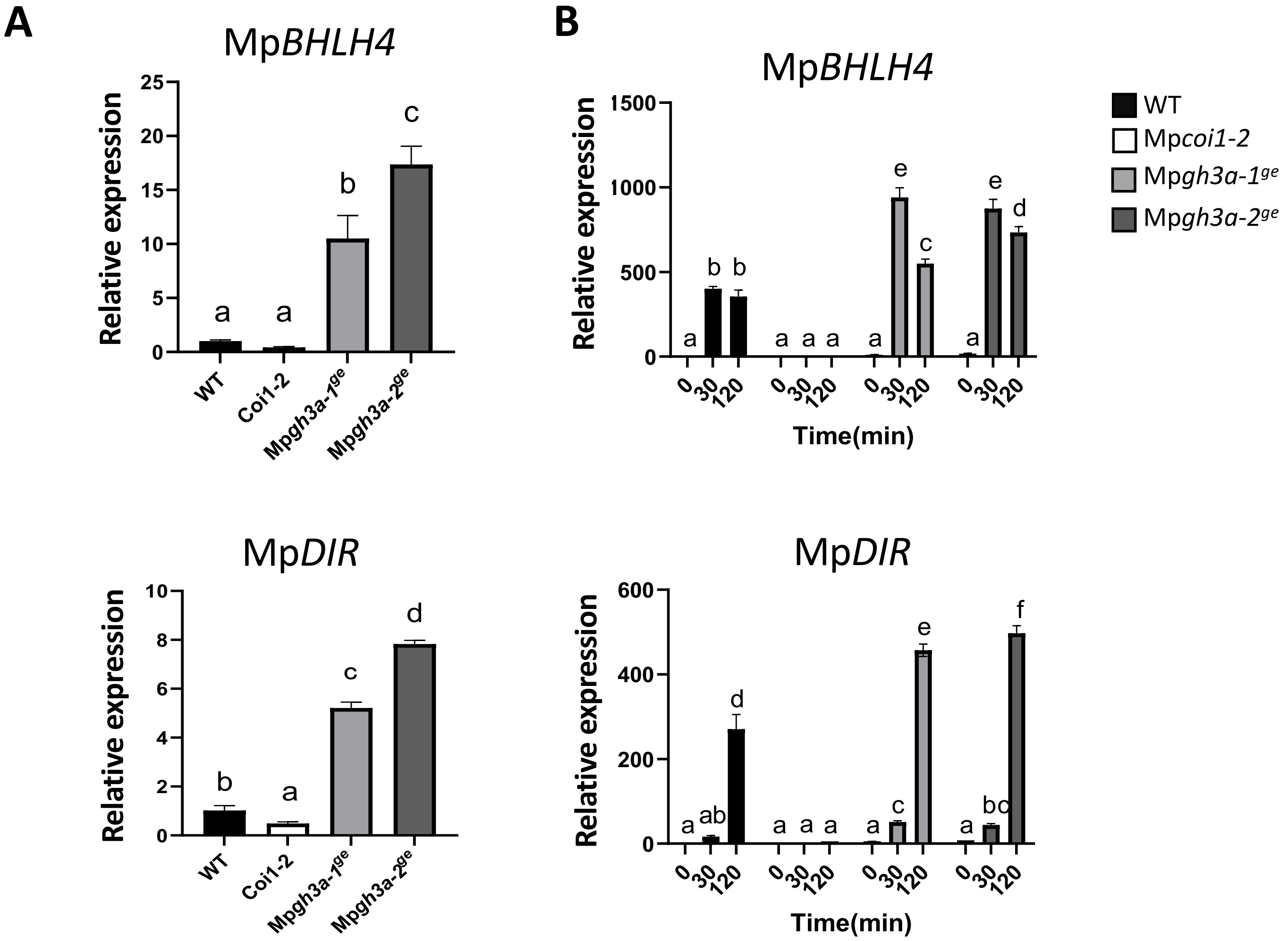

Supplementary Figure S5. Expression of dn-OPDA marker genes in Mp*gh3a*^{ge} mutant plants. RT-qPCR analysis of OPDA-regulated genes in wild-type Tak-1 (WT), Mp*coi1-2*, Mp*gh3a-1*^{ge} and Mp*gh3a-2*^{ge} plants in response to wounding (A-B). Untreated plants (0) were included as control (A). Plants were collected after the indicated times (B). Expression of the Mp*BHLH4* (Mp*2g00930*) and Mp*DIR* (Mp*5g16510)*) genes was normalized against Mp*ACT* (Mp*6g11010*). Data shown as mean ± s.d. of three biological replicates, 3 plants per each replicate. Experiments were repeated three times with similar results. Letters indicate significant different samples according to the one-way ANOVA/Tukey HSD post hoc test ($P < 0.05$).

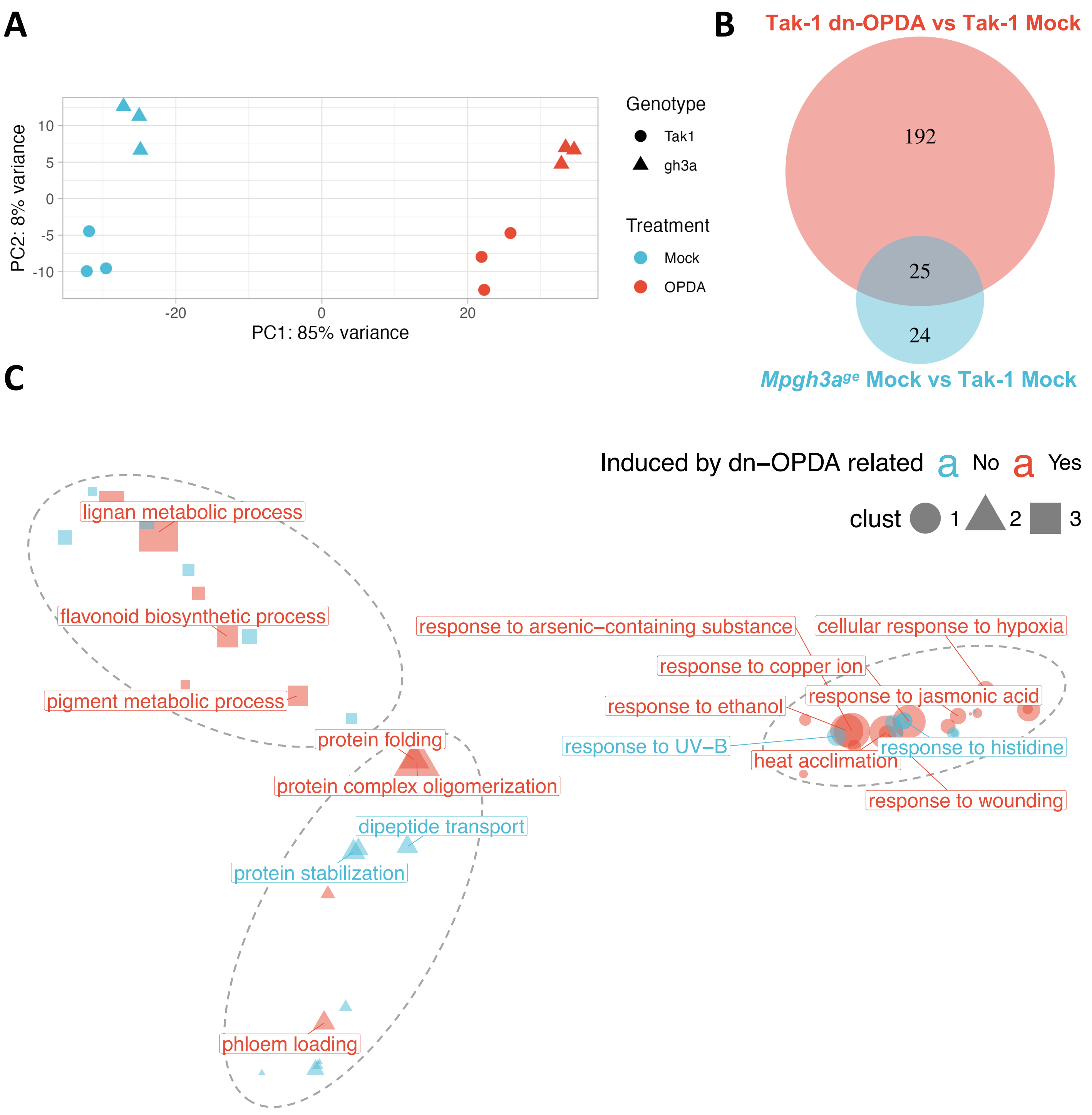

Supplementary Figure S6. Transcriptomic analysis of *Mpgh3a-1*^{ge} in basal conditions.

(A) Principal Component Analysis (PCA) plot of the 1000 top genes.

(B) Venn diagram of enriched Gene Ontology (GO) terms representing up-regulated DEGs after dn-OPDA treatment in Tak-1 (red) and GO terms representing up-regulated DEGs in *Mpgh3a-1*^{ge} compared to Tak-1 in Mock conditions (blue).

(C) PCA plot of Gene Ontology (GO) terms based on their score, representing up-regulated DEGs in Tak-1 after dn-OPDA treatment compared to Tak1 in Mock conditions. Each cluster is identified with a different shape and delimited by a dashed circle. GO terms induced by OPDA treatment in WT pants are highlighted in red.

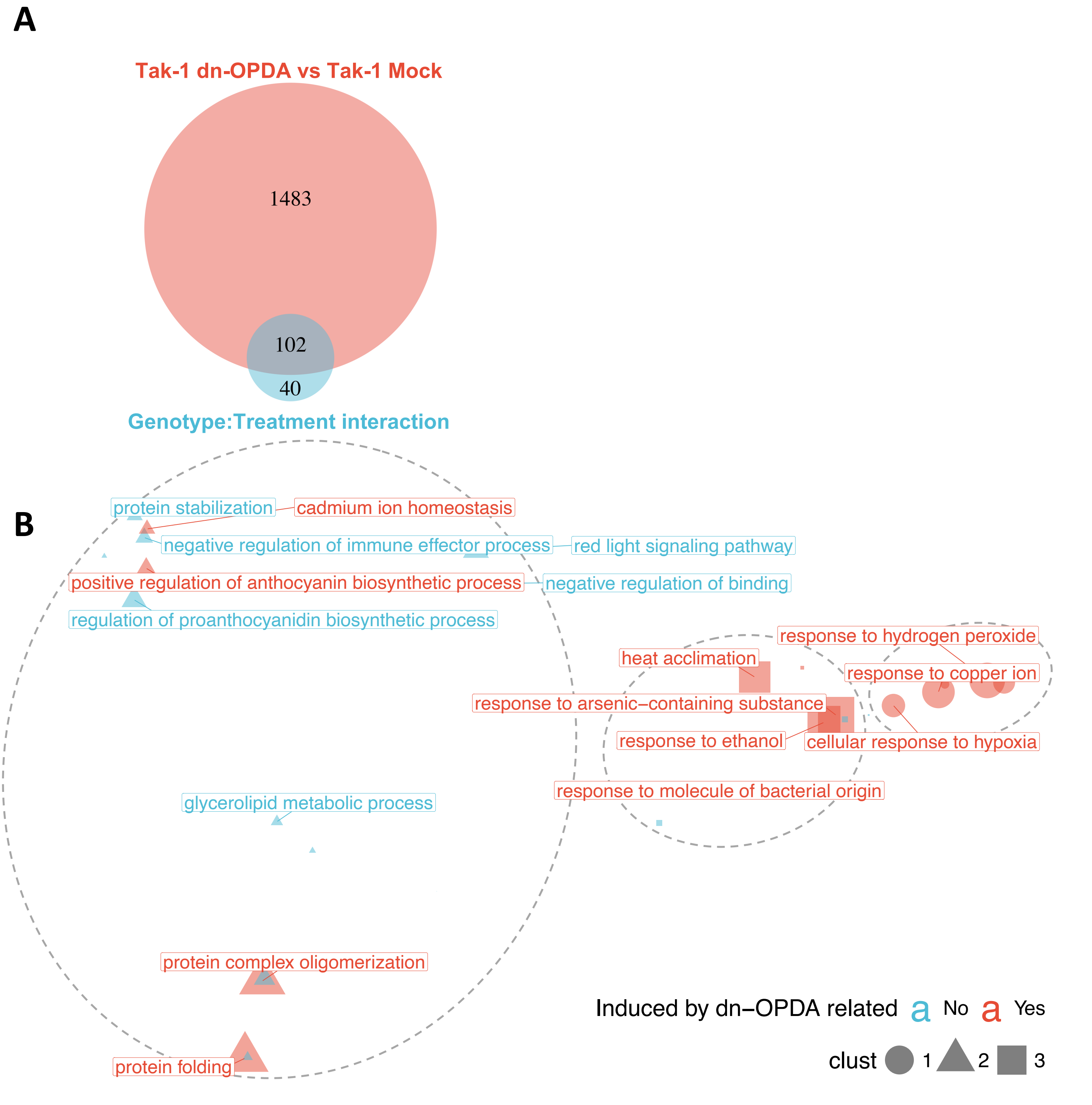

Supplementary Figure S7. Gene Ontology and WGCNA analyses of *Mpgh3a-1*^{ge} in response to OPDA treatment.

(A) Venn diagram of differentially expressed genes (DEGs) up-regulated after dn-OPDA treatment in Tak-1 (red) and DEGs from the genotype:treatment interaction (blue).

(B) PCA plot of Gene Ontology (GO) terms based on their score, representing 117 DEGs from turquoise module generated by Weighted Gene Co-expression Network Analysis (WGCNA) after using DEGs from the genotype:treatment interaction effect. Each cluster is identified with a different shape and delimited by a dashed circle. GO terms induced by OPDA treatment in WT pants are highlighted in red.

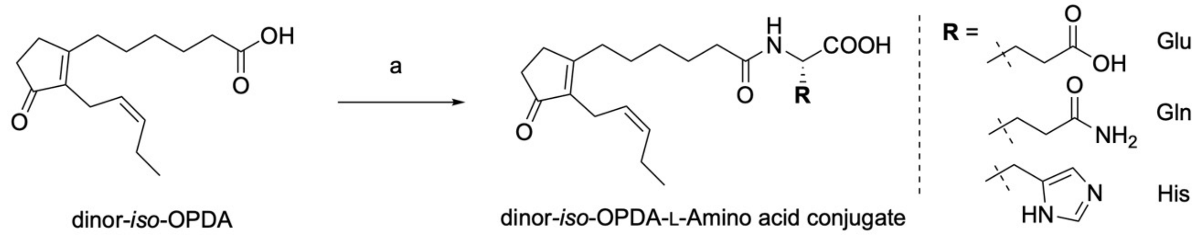

Supplementary Figure S8. Synthetic scheme of dinor-*iso*-OPDA-L-Amino acid conjugates. Schematic representation of the chemical reaction for the synthesis of dinor-*iso*-OPDA-L-Amino acid conjugates. Reagents and conditions: (a) $ClCO_2Et$, Et_3N, THF, 0 °C; L-amino acid, DIPEA, H_2O.

Supplementary Table S3. List of primers used in this work.

Primer Name	Sequence
MpGH3A gRNA 1A	ctcgTTTCGCCTCTGCCGATTCTC
MpGH3A gRNA 1B	aaacGAGAATCGGCAGAGGCGAAA
MpGH3A gRNA 2A	ctcgCAAAGCTCTCGGGGATAAGA
MpGH3A gRNA 2B	aaacTCTTATCCCCGAGAGCTTTG
MpGH3A gRNA 3A	ctcgACGAGCGCTTGAGAACACCG
MpGH3A gRNA 3B	aaacCGGTGTTCTCAAGCGCTCGT
MpGH3A gRNA 4A	ctcgTGTCGAGGTCCTATTTCAAG
MpGH3A gRNA 4B	aaacCTTGAAATAGGACCTCGACA
MpGH3A Genotyping Fw	TTAGGTGGTGGAGAGGGGAG
MpGH3A Genotyping Rv	GAAGGGCCTCTTCTGGAACC
MpGH3A qPCR Fw	ATTCTCGTCACCACCGTGTC
MpGH3A qPCR Rv	TATCGATCGACAGCACCGTG
MpActin qPCR Fw	AGGCATCTGGTATCCACGAG
MpActin qPCR Rv	ACATGGTCGTTCCTCCAGAC
MpDIRIGENN qPCR Fw	CGGAGAAGGTAATTGTCACCACA
MpDIRIGEN qPCR Rv	TCTACCATACAGAGGACGTGATCG
MpBHLH4 qPCR Fw	AGTACGTAAAACAGCTGAGATCACG
MpBHLH4 qPCR Rv	GGTTAATGCTCTCCAACTCCTGA
